# miRNA-383-5p Regulated Migration and Invasion of Tumor Cells by Inhibiting NCKAP1 Expression in Gastric Cancer

**DOI:** 10.1007/s10528-024-10804-7

**Published:** 2024-04-16

**Authors:** Chen Wang, Pan Wang, Yuan Tian, Cuijuan Lu, Lixia Liu, Jianguo Wu, Yanan Wang, Jinghua Li

**Affiliations:** 1 Department of Pathology, Affiliated Hospital of Hebei University, No. 212 East Yuhua Road, Baoding, Hebei Province 071000 China; 2 Department of No.2 Gastroenterology, Affiliated Hospital of Hebei University, Baoding, China; 3 Department of Ultrasound, Affiliated Hospital of Hebei University, Baoding, China; 4 Department of Information Center, Affiliated Hospital of Hebei University, Baoding, China; 5 Department of Surgery, Affiliated Hospital of Hebei University, Baoding, China

**Keywords:** Gastric cancer, miR-383-55p, NCKAP1, Migration, Invasion

## Abstract

Gastric cancer (GC) is the second deadliest disease in Asia, so it is crucial to find its promising therapeutic targets. The expression profile data of miR383-5p in the Cancer Genome Atlas (TCGA) were analyzed. The expression levels of miR383-5p in the collected clinical tissue samples and peripheral blood samples were examined by qPCR, and the relationship between its expression and the clinical data of patients was evaluated. MiR383-5p was overexpressed in the AGS cells, and cell biology assays, such as Transwell, were performed to detect the cell proliferation, migration, invasion and other cell biology abilities of miR383-5p. Target prediction and dual luciferase reporter gene assay were performed to find and validate the target genes of miR383-5p. The expression and activity of MMP and related proteins after overexpression of miR383-5p and NCKAP1 were detected by WB and gelatin zymography assay. The expression of miR383-5p was down-regulated in GC tissues, and its low expression was associated with lymph node metastasis. Restoration of miR383-5p expression in GC cells can inhibit the invasion and migration abilities of GC cells. MiR383-5p negatively regulated NCKAP1 through direct interaction with the 3’UTR sequence of NCKAP1. The overexpression of NCKAP1 can improve the migration and invasion abilities of GC cells, whereas overexpression of miR383-5p can inhibit growth of the aforementioned abilities of GC cells induced by NCKAP1 overexpression. The overexpression of NCKAP1 can increase the expression level and activity of MMP2, while the overexpression of miR383-5p can inhibit the increase of MMP2 expression level and activity in GC cells induced by NCKAP1 overexpression. NCKAP1 is a target gene of miR383-5p, and miR383-5p could be a valuable therapeutic target for stomach adenocarcinoma.

## Introduction

Gastric cancer (GC) is the third leading cause of cancer-related deaths and the second deadliest disease in Asia (Bray et al. 2018). Advanced GCs generally involve poor therapeutic effects and short survivals, and the reason is often the migration of metastatic GC cells to distant tissues and lymph nodes (Thrumurthy et al. 2015). Although there are multiple standard treatments such as surgery, endoscopic mucosal resection and chemotherapy, and all are widely used, factors such as primary resistance or acquired resistance ultimately lead to treatment failure and poor prognosis of GC patients. To address these problems, new therapeutic approaches, such as targeted therapy and immunotherapy, have gained tremendous attention and achieved significant development in recent years (Digklia and Wagner 2016).

As the most studied ncRNAs, miRNAs have been shown to be involved in almost all cellular functions, such as proliferation, apoptosis, epithelial-mesenchymal transition (EMT), autophagy and cell cycle control. A variety of miRNAs act as oncogenes or tumor suppressor genes during carcinogenesis, which can also be used as the diagnostic and prognostic markers for cancer patients after certain treatments (Guo et al. 2019; Liu et al. 2019). miRNA can bind to the 3’-untranslated region (3’-UTR) of multiple target genes, thus repressing gene expression after transcription (Shukla et al. 2011). Studies show that multiple miRNAs have abnormal expressions in gastric cancer, and are involved in the development and progression processes of GC (Stuart-Smith and Vanhoutte 1988; Wang et al. 2018). Zhu et al. found that miR-106 can inhibit apoptosis of GC cells by targeting the JAK/STAT signaling pathway, and promoted the invasion and metastasis of GC (Zhu et al. 2019). miR-135a can inhibit the migration of GC cells by targeting tumor necrosis factor receptor-associated factor 5 (TRAF5) and its downstream nuclear factor kappa-B (NF-Κb) pathway. Moreover, low expression of miR-135a in GC tissues and cell lines predicted poor overall survival, suggesting its diagnostic value as a biomarker in gastric cancer. (Xie et al. 2019).

MicroRNA-383 (miR-383) has emerged as a significant regulator in various pathological conditions, including tumor progression and male infertility. In male infertility, particularly in cases of maturation arrest, miR-383 expression is notably reduced(Lian et al. 2010). MiR-383 exerts its effects by targeting interferon regulatory factor-1 (IRF1), a tumor suppressor, leading to inhibition of proliferation through G1-phase arrest and induction of apoptosis. Additionally, miR-383 downregulates cyclin-dependent kinase 4 (CDK4), further contributing to cell cycle arrest and inhibition of retinoblastoma protein (pRb) phosphorylation(Tian et al. 2013). In gliomas, miR-383 targets insulin-like growth factor 1 receptor (IGF1R), leading to suppression of AKT signaling and downregulation of matrix metalloproteinase 2 (MMP2), thereby inhibiting glioma cell invasion. Its downregulation correlates with higher pathological grades in gliomas(He et al. 2013). These findings suggest that miR-383 could serve as a potential diagnostic marker or therapeutic target in glioma treatment.

The NCK-associated protein 1 (NCKAP1) gene encodes the NCKAP1 protein, which connects the cytoskeleton and signaling pathways in cells (Xiong et al. 2019). In breast cancer, the overexpression of NCKAP1 is associated with poor prognosis, indicating that it may play a role in the malignancy and prognosis of breast cancer (Teng et al. 2016). Furthermore, NCKAP1 is also found to be associated with the cell migration and invasion processes (Xiong et al. 2019). Cell migration and invasion are important features of tumor metastasis, which play an important role in tumor development and metastasis to other tissues and organs. Therefore, the abnormal expression of NCKAP1 may have an effect on the processes of tumor metastasis and invasion. At present, there have been no reports on the relationship between NCKAP1 and gastric cancer.

In this study, we analyze the expression profile data of miR383-5p in the Cancer Genome Atlas (TCGA), and found that its expression level in Stomach Adenocarcinoma (STAD) tissues was lower than that in normal tissues. The results of cell biology experiments (such as Transwell) showed that overexpression of miR383-5p can inhibit the invasion and motility of stomach adenocarcinoma cells, but has no effect on their proliferation. The results of target gene prediction and dual luciferase reporter gene assay showed that NCKAP1 is the target gene of miR383-5p. In summary, miR383-5p could be a potential therapeutic target for stomach adenocarcinoma.

## Material and Method

### TCGA Database Analysis

The expression data of miR383-5p in stomach adenocarcinoma from the TCGA database were obtained online using the UALCAN data portal (https://ualcan.path.uab.edu/))(Chandrashekar et al., 2022).

### Specimen Collection

98 cases of GC samples and their matched paracancerous tissues, and peripheral blood came from the Affiliated Hospital of Hebei University (May, 2020 to Mar, 2023). All patients signed a written informed consent before surgery. This study was approved by the Ethics Committee of the Affiliated Hospital of Hebei University.

### Cell Lines and Cell Culture

The human STAD cell line AGS was purchased from the National Infrastructure of Cell Line Resource. Both AGS and SGC-7901 were cultured using DMEM (Thermo Fisher Scientific, USA) complete medium containing 10% FBS (Thermo Fisher Scientific, USA). All cells were incubated and cultured at 37 °C in 5% CO_2_.

### Lentivirus Construction and Transfection

The pLV-has-miR383-5p and pLV-miRNA-NC plasmids were obtained from Biosettia Inc. (San Diego, CA, USA); the NCKAP overexpression vector pcDNA3.1-NCKAP was obtained from GeneChem Co., Ltd. (Pudong, Shanghai, China). The production and infection of retrovirus were performed as described previously (Deng et al. 2013). In short, pLV-has-miR383-5p or pcDNA3.1-NCKAP and packaging plasmid were transfected into the 293T cells using the Lipofectamine 2000 reagent (Invitrogen), and after incubation for 48 h, the virus particles were harvested for infection of GC cells.

### Fluorescence Quantitative PCR

Total RNA was extracted from cells using the TRIzol reagent (Invitrogen, USA) according to the manufacturer’s instructions. Peripheral blood samples was extracted using the miRNeasy Serum/Plasma Kit (Cat. No.217,184, QIAGEN, Germany). The purity and concentration of total RNA were determined by UV spectrophotometry. Then, cDNA was synthesized using SuperScript III (Invitrogen) according to the manufacturer’s instructions. See Table 1 for the primer sequences. In the ABI PRISM 7000 Fluorescent Quantitative PCR System (Applied Biosystems, Foster City, CA, USA), the standard SYBR Green PCR kit (Takara, Dalian, China) was used to perform qPCR. qPCR procedure: degeneration (at 95 °C for 5 s), annealing (at 55 °C for 30 s) and extension (at 72 °C for 30 s), and it was repeated for 40 cycles. The relative expressions of genes were analyzed using the 2^−ΔΔCt^ method (Livak and Schmittgen 2001). The miRNA internal reference is U6smiRNA.

**Table 1:** The Primer Sequences

miRNA/Gene	Primer sequences 5’-3’
miR383-5p	qF-TCAGAAGGTGATTGTGGCTAAA
U6s miRNA	qF-GAGGGCCTATTTCCCATGATT
NCKAP1	qF-CAATCACCTTCTTCTCAG
	qR-TTCTTCACTCCTCATCTT
β-Actin	qF-CTCTTCCAGCCTTCCTTCCT
	qR-AGCACTGTGTTGGCGTACAG

### Western Blotting Analysis

After collecting the transfected cells, they were washed using PBS (Solarbio, Beijing, China). The supernatant was collected after lysis to obtain the total protein. The protein concentration was determined using the BCA protein assay kit (Solarbio, Beijing, China). After separation using 12% SDS-page (Beyotime, Shanghai, China), the cells were transferred to a PVDF membrane (Thermo Fisher, Carlsbad, CA, USA). The membrane was closed for 1 h at room temperature using the Tris-Buffered saline (Solarbio, Beijing, China) containing 5% skim milk powder. Next, the cells were incubated overnight with rabbit anti-NCKAP1 antibody (1:1000, ab126061, Abcam, Shanghai, China) or β-Actin (1:5000, ab179467, Abcam, Shanghai, China). On the following day, after rinsing with TBST, the cells were incubated with goat anti-rabbit IgG H&L (HRP) (1:2000, ab205718, Abcam, Shanghai, China) for 1 h at room temperature, and images were obtained using an infrared laser scanning imaging system (CDYSSEY CLx; General Electric).

### Cell Proliferation Assay

Cell proliferation assay was performed using Cell Light EdU Apollo 567 In Vitro kit (# C10310, Guangzhou Ruibao Biological Co., Ltd.) according to the manufacturer’s instructions. The EdU solution was added to routinely cultured cells and incubated for 2 h. After fixing the cells with cell fixative, glycine was added for incubation. Then, after the cells were washed using PBS, they were incubated with osmotic agent containing 0.5% TritonX-100. Next, after using the 1 × Apollo dye solution to incubate the cells, the penetrant was added for incubation. Then, the DAPI dye reaction solution was added to stain the cells. Five 200 × fields of view were randomly selected for observation and photo shooting under a fluorescence microscope.

### CCK-8 Assay

The cell viability assay was perfomed using Cell Counting Kit-8 according to the instructions: the cells were inoculated in a 96-well plate with a cell density of approximately 2 × 10^3^cells/well, then, 10 µL CCK-8 solution was added to the cells to prevent bubbles at 0 h, 24 h, 48 h, 72 h, and the cells were incubated in a 5% CO_2_ incubator at 37 °C for 2 h. The absorbance at 450 nm was measured using an enzyme meter. Cell viability (%) = [ (As-Ab) / (Ac-Ab)] × 100%, As = absorbance of the experimental wells, Ab = absorbance of blank wells, Ac = absorbance of the control wells.

### Transwell Assay

As per the manufacturer’s instructions, Invasion experiments were performed using Matrigel (BD Biosciences, San Jose, CA, USA) coated Transwell chamber (Corning, Corning, NY, USA). Then, 1 × 10^5^ suspended serum-free transfected cells were placed into the upper chamber be paved with Matrigel, and the lower chamber was filled with 10% FBS DMEM as chemoattractant. The chambers were placed in a 37℃ incubator and incubated for 24 h. After removing the upper chamber from the lower chamber, the lower chamber was stained using crystal violet after fixation with 4% paraformaldehyde. Photographs were taken in six separate cell areas and counted on filter.

### Wound Healing Assay

The cell migration ability was tested using the wound healing assay. In the assay, 1 mL of cell suspension with a density of about 1 × 10^5^ was inoculated into a 6-well plate, and the culture medium was added to form a monolayer of cells after wall attachment. A 10 µL sterile tip was used to scratch vertically on the monolayer cells, and the separated cells were washed with PBS. The cells were incubated in an incubator containing 5% CO_2_ at 37 °C. The cells were observed and photographed at 0 h and 24 h, respectively. In order to reduce the influence of cell proliferation on the migration results, the serum-free medium was used in the wound healing assay, and the cells were treated with 1 µg/ml mitomycin for 1 h to inhibit cell division.

### Luciferase Assay

The 3’-UTR region of NCKAP1 and its mutant sequences were cloned into pGL4.13 [luc2/SV40] vector (Promega) to construct a reporter gene vector. The human embryonic kidney (HEK293) cells were co-transfected with miR383-5p mimic or mimic-NC with firefly luciferase reporter gene vector and Renilla luciferase vector. Then, 48 h after co-transfection, the Dual-Luciferase® Reporter Assay System (E1910, Promega, Madison, WI, USA) and the Glomax 96 microplate spectrophotometer (Promega, Madison, WI, USA) were used to determine the activities of firefly luciferase and Renilla luciferase.

### Gelatin Zymography for Detection of MMP Activity

After collecting the cells, they were washed using PBS (Solarbio, Beijing, China). The supernatant was collected after lysis to obtain the total protein. The protein concentration was determined using the BCA protein assay kit (Solarbio, Beijing, China). Electrophoretic separation was performed using SDS- PAGE containing 0.1% gelatin (Beyotime, Shanghai, China), and the gel was rinsed after elution in eluent (2.5% Triton X-100, 50 mM Tris-HCl, 5 mM CaCl_2_, 1 µM ZnCl_2_, pH 7. 6), and subsequently placed in the incubation solution (50 mM Tris, pH 7.5, 150 mM NaCl, 10 mM CaCl_2_, 1 µM ZnCl_2_, 0.02% Brij-35) at 37 °C for 42–48 h. The gel was stained with CBB (Coomassie brilliant blue), and the intensity of the bands could be observed after decolorization.

### Statistical Analysis

GraphPad Prism 9 (GraphPad Software; Dotmatics) was used. All data are expressed in terms of mean ± standard deviation. Differences between groups were statistically analyzed using one-way ANOVA and Tukey post hoc tests. The differences were considered statistically significant when *P* < 0.05.

## Results

### miR383-5p was Downregulated in STAD

**Fig. 1:**
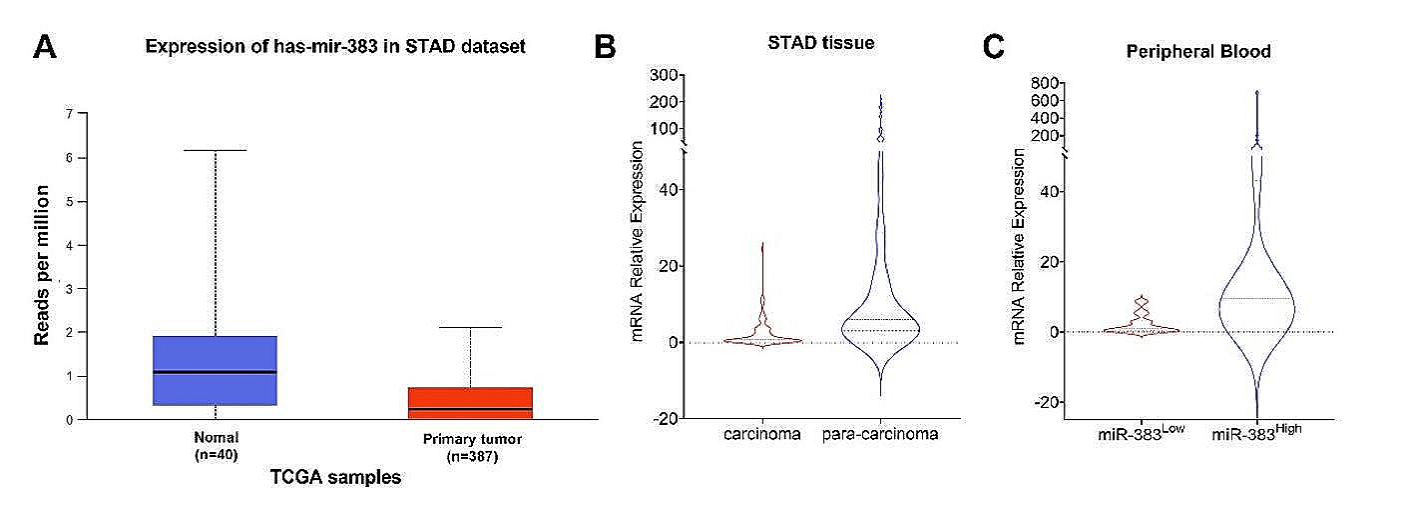
miR383-5p expression is down-regulated in STAD **A** Data in TCGA database show down-regulated miR383-5p expression in STAD; **B** miR383-5p expression in clinical samples; **C** miR383-5p expression in peripheral blood

The expression profiling results of data from the TCGA database showed that miR383-5p was significantly down-regulated in STAD compared with normal tissues (Fig. 1A); we collected the clinical tissue samples and peripheral blood samples from 98 cases of STAD, and the general characteristics of the patients are shown in Table 2. The miR383-5p expression in 98 collected cases of STAD and their paracancerous tissues was examined by qPCR, and the results showed that the expression level of miR383-5p in STAD was significantly lower than that in paracancerous tissues (Fig. 1B). Then, we examined the miR383-5p expression in the peripheral blood of the patients, divided them into miR383-5p high expression group (miR-383^High^) and miR-383 low expression group (miR-383^Low^) according to the median (Fig. 1C), and assessed the correlation between the miR383-5p expression and the degree of tumor metastasis (Table 2). The results showed that the occurrence and degree of metastasis in the miR-383^Low^ group were significantly higher than that of the miR-383 ^High^ group, suggesting that there is a correlation between stomach adenocarcinoma tumor metastasis and down-regulation of miR383-5p expression.

**Table 2:** Relationship between miR383-5p expression and clinical characteristics in stomach adenocarcinoma

Characteristic		n	miR383-5p expression, n		P
			High	Low	
Total		98	53	45	
Gender	Male	52	27	25	0.6884
	Female	46	26	20	
Age	≤ 60	58	28	30	0.2165
	> 60	40	25	15	
Grade	I-II	45	24	21	ns
	III-IV	53	29	24	
Nodal metastasis status	N0-N1	63	39	24	0.0194
	N2-N3	35	14	21	

### Overexpression of miR383-5p Inhibited STAD Invasion and Migration

**Fig. 2:**
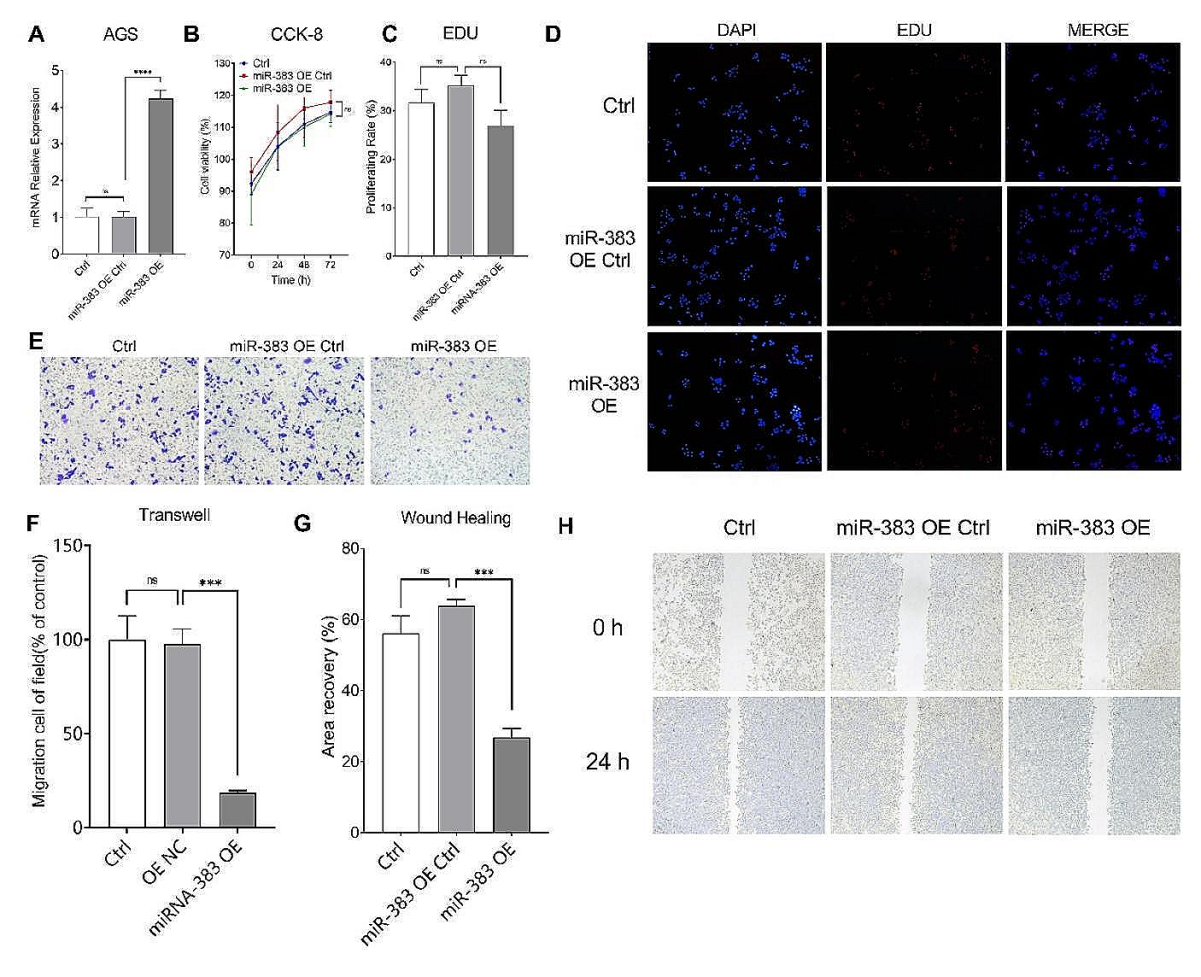
Cell biological function assay of miR383-5p overexpression cell line. **A** qPCR verified that miR383-5p overexpression cell line was successfully constructed; **B** CCK8 results showed that miR383-5p overexpression had no significant effect on cell viability; **C** EDU results showed that miR383-5p overexpression had no significant effect on cell proliferation amplification, 10 ×; **D** Representative images of EdU staining; **E** Representative images of Transwell, amplification, 10 ×; **F** Transwell results show that miR-383 overexpression inhibited cell invasion; **G** Wound healing assay results show that miR-383 overexpression inhibits cell migration; **H** Representative images of Wound healing assay, amplification, 10 ×. * *p <* 0.05; ** *p <* 0.01; *** *p <* 0.001

We used AGS to construct the miR383-5p overexpression cell line (miR383-5p OE) (Fig. 2A), and tested the effect of miR383-5p overexpression on the viability of GC cells by CCK8 assay. According to the results as shown in Fig. 2B, there was no significant difference in cell viability between the miR383-5p OE group and the control group. The results of EdU assay of cell proliferation ability are shown in Fig. 2C&D. There was no significant difference in the proliferation ability of cells between the miR383-5p OE group and the control group, i.e., overexpression of miR383-5p did not have a significant effect on the proliferation ability and viability of GC cells. Subsequently, we performed the Transwell assay to detect the invasive ability of the cells. The results, as shown in Fig. 2E&F, indicated that the number of transmembrane cells in the miR383-5p OE group was significantly less than that of the control group. Similarly, the results of the wound healing assay showed that the wound healing rate in the miR383-5p OE group was significantly lower than that of the control group (Fig. 2G&H). The above results indicated that overexpression of miR383-5p inhibited the invasion and migration ability of GC cells, suggesting that down-regulated miR383-5p in GC tissues may be involved in the metastasis of tumor tissues.

### NCKAP1 is a Direct Functional Target of miR383-5p in STAD

**Fig. 3:**
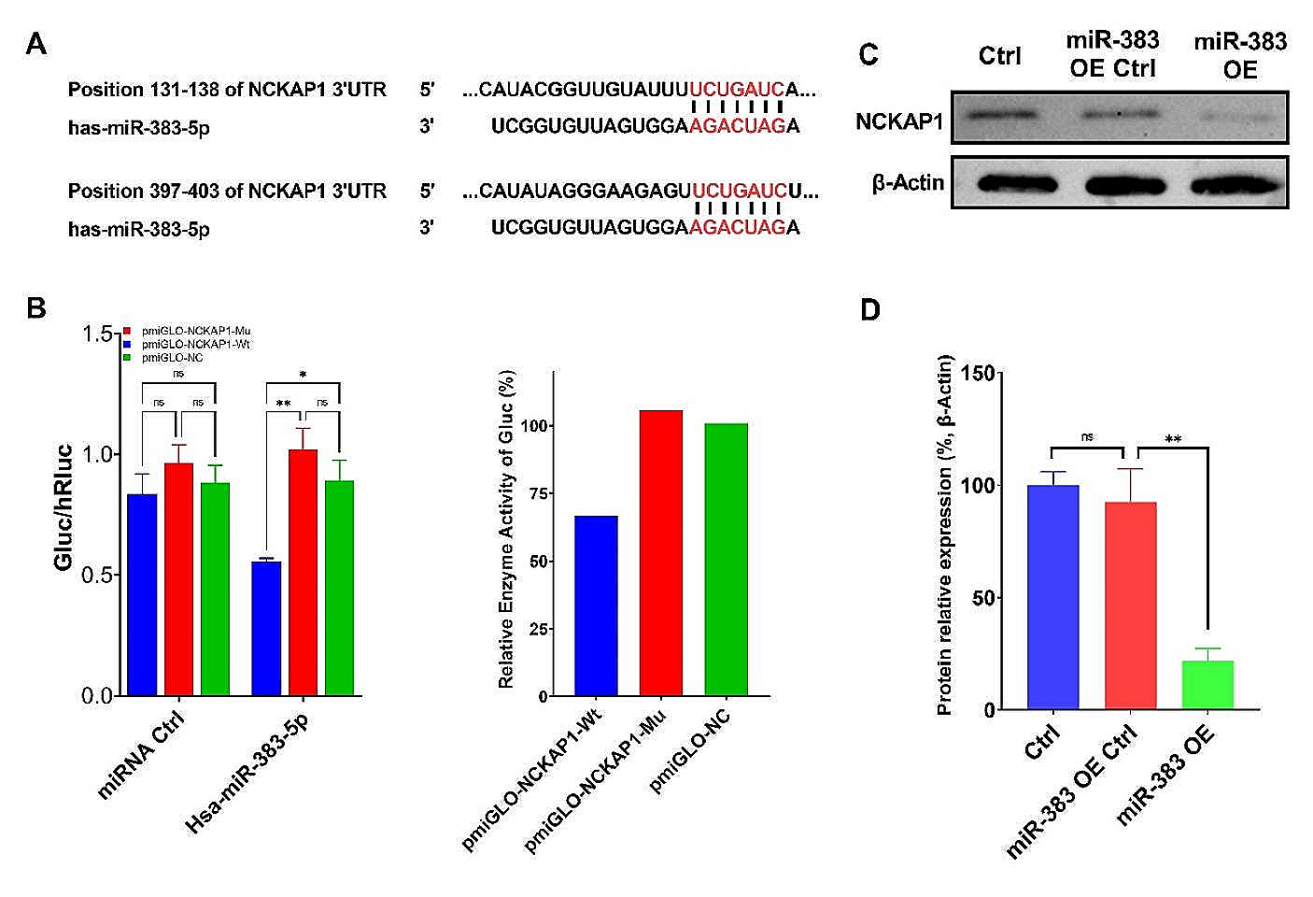
Verification of targeting relationship between miR383-5p and NCKAP1. **A** miR383-5p had targeting binding sequenced with NCKAP1; **B** Dual luciferase assay showed that miR-383-5p could bind to NCKAP-Wt; **C** & **D** WB assay showed that miR383-5p overexpression inhibited the protein expression of NCKAP1 in GC cells. * *p <* 0.05; ** *p <* 0.01

It was predicted that miR383-5p had target binding sequences with NCKAP1 by TargetScan-Human (http://www.targetscan.org) (Fig. 3A). Then, we performed the dual luciferase reporter gene assay to verify the targeting relationship between the two. According to the results in Fig. 3B, the fluorescence intensity of the NCKAP1-WT group was significantly down-regulated, i.e., miR383-5p only bound to the correct 3’-UTR region of the NCKAP1 gene, suggesting that NCKAP1 is the target gene of miR383-5p. The protein expression of NCKAP1 in the GC cells with overexpressed miR383-5p was detected by WB, and the results were shown in Fig. 3C&D. Compared with the control group, NCKAP1 was significantly down-regulated in the miR383-5p mimic group.

### Overexpression of miR383-5p Inhibited the Expression of NAKAP1 in STAD Cell Lines

**Fig. 4:**
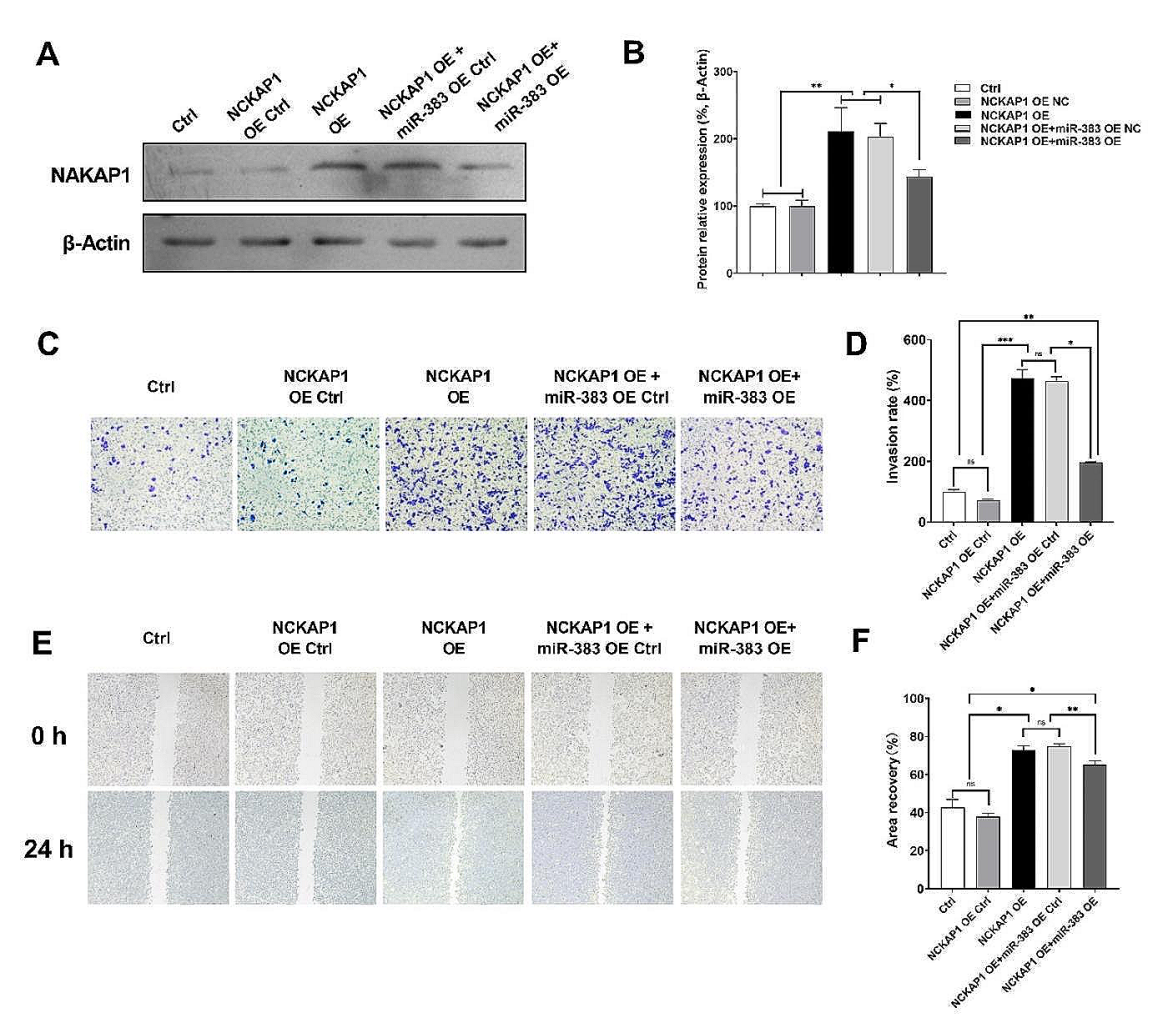
miR383-5p affected some biological functions of STAD cell lines by inhibiting NAKAP1 expression. **A**&**B** Overexpressed miR383-5p inhibited the expression of NAKAP1; **C** & **D** Transwell results indicated that overexpression of miR383-5p can inhibit to a certain extent the increase in invasive ability of gastric cancer cells caused by overexpression of NAKAP1, amplification, 10 ×; **E** & **F** The wound healing assay results indicated that overexpression of miR383-5p can inhibit to a certain extent the increase in migratory ability of gastric cancer cells caused by overexpression of NAKAP1, amplification, 10 ×. * *p <* 0.05; ** *p <* 0.01; *** *p <* 0.001

Next, we constructed the STAD cell line with simultaneous overexpression of both NCKAP1 and miR383-5p, and examined their cellular biological functions. The WB results showed that after simultaneous overexpression of miR383-5p and NAKAP1 (NCKAP1 OE + miR383 OE), the up-regulation of NCKAP1 expression was significantly inhibited compared with overexpression of NCKAP1 alone (NCKAP1 OE) (Fig. 4A&B). The results of Transwell assay and wound healing assay indicated that overexpressed NAKAP1 could promote the invasion and migration ability of GC cells, while miR383-5p could inhibit the increase in the invasion and migration ability of GC cells caused by NAKAP1 overexpression to certain extent (Fig. 4C-F).

**Fig. 5:**
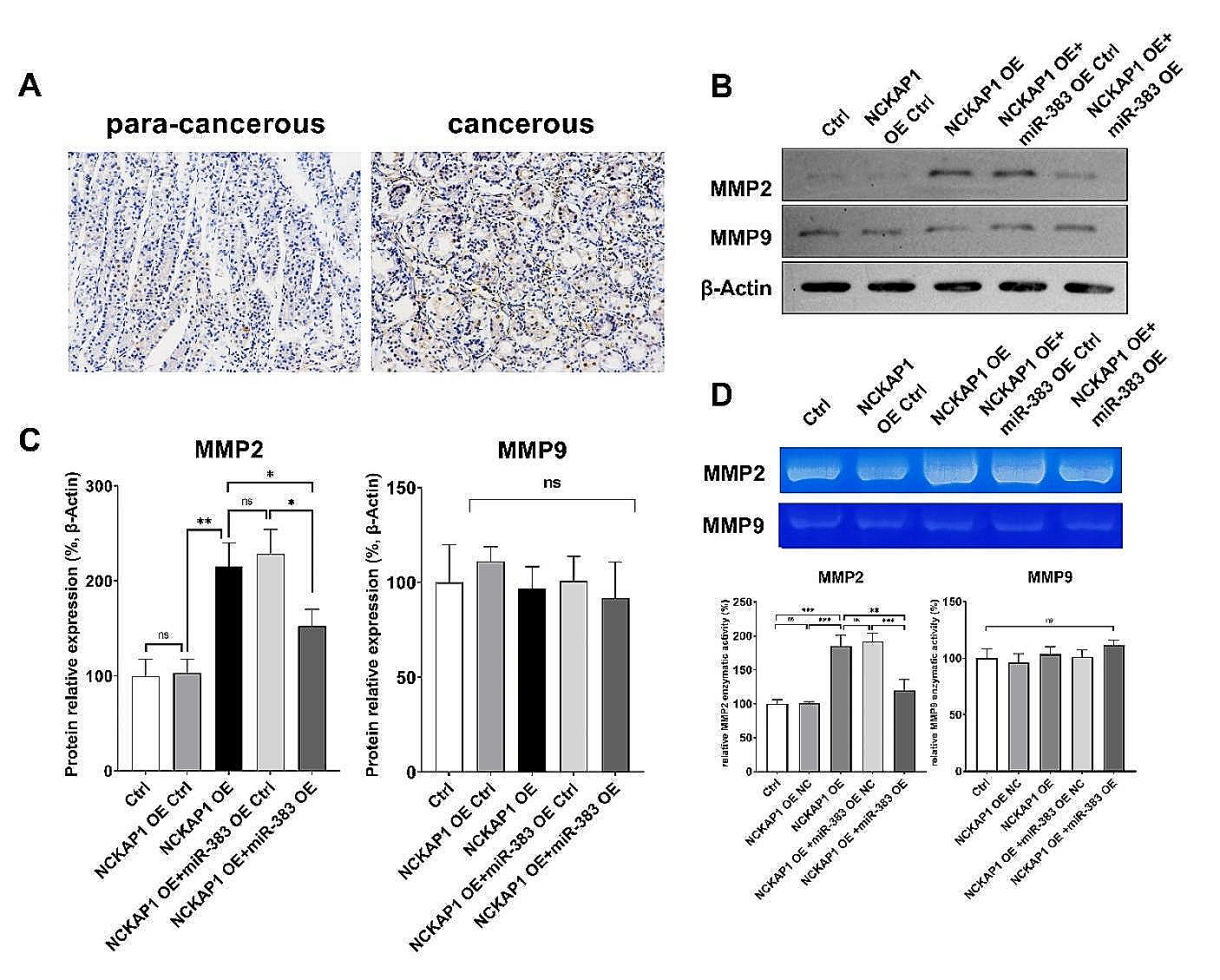
Activated MMP2 is involved in the process of miR383-5p-NCKAP1 affecting the motility of STAD cells **A** IHC results showed that the expression level of NCKAP1 in STAD tissues was significantly higher than that in paracancerous tissues, amplification, 10 ×; **B** & **C** WB assay showed that miR383-5p could inhibit the increased expression of MMP2, but not MMP9, caused by the overexpression of NCKAP: **D** Gelatin zymography assay showed that miR383-5p could inhibit the increased activity of MMP2 caused by NCKAP overexpression, but not MMP9. * *p <* 0.05; ** *p <* 0.01; *** *p <* 0.001

Then, we examined the expression level of NCKAP1 in the collected STAD samples and the paracancerous tissues by IHC, and the results showed that the expression level of NCKAP1 was significantly higher in the STAD tissues than in the paracancerous tissues (Fig. 5A). Sufficient evidences suggest that the metastatic ability of tumor is closely related to its production or induction of basement membrane proteins that degrade the extracellular matrix (ECM), and there are more relevant studies on metalloproteinases (MMP) and gastric cancer(Bashash et al. 2013). Activated MMP is involved in ECM remodeling (Brew and Nagase 2010), which is involved in tumorigenesis, angiogenesis and distal metastasis. Therefore, we examined the expression levels of MMP2 and MMP9 in the miR383-5p OE and NCKAP1OE cell lines by WB. The results are shown in Fig. 5B/ According to the results, the secretion of MMP2 was significantly increased in the NCKAP1 OE cells compared with the blank control, while the expression of MMP2 was decreased in cells that also had overexpressed miR383-5p. Next, we examined the MMP activity by gelatin zymography assay. According to the results as shown in Fig. 5C, the intensity of MMP2 bands in NCKAP1 OE cells was significantly higher than that in the control group, and the intensity of the bands was significantly weakened after the simultaneous overexpression of miR383-5p. In other words, the overexpression of NCKAP1 in GC cells could increase the MMP2 activity, whereas miR383-5p could inhibit the induction of increased MMP2 activity by NCKAP1. The above results suggest that the changes of miR383-5p affecting the motility of STAD cells through NCKAP1 may be mediated by the increase of MMP2 expression and activity.

## Discussion

Related studies show that miRNAs regulate more than one-third of human genes (Lewis et al. 2005), and are involved in the regulation of a variety of biological processes, including cell growth, apoptosis, metabolism and transformation (Lu and Rothenberg 2018; Rottiers and Naar 2012). The expression profiling shows that many miRNAs are downregulated in cancer samples compared to corresponding normal tissues, while some are overexpressed (Pang et al. 2009). A global miRNA analysis in medulloblastoma (MB) showed that the miR383-5p expression was down-regulated in MB, whereas the miR383-5p abundance was high in normal brain tissue (Li et al. 2013; Liang et al. 2007). The ectopic expression of miR-3 resulted in cell growth inhibition, altered cell cycle distribution, enhanced apoptotic cells and altered expression of apoptosis-related proteins (Li et al. 2013). In our study, miR383-5p was found to be down-regulated in GC tissues based on analysis of the TCGA data. After overexpressing miR383-5p in GC cells by in vitro cellular experiment, the invasion and migration abilities of GC cells were significantly inhibited. However, different from the previous studies, the proliferative ability of GC cells did not show no significant change compared with control after overexpression of miR383-5p in our study.

NCKAP1 is a protein associated with the Src homology 3 (SH3) structural domain of NCK proteins, which mediates contact-dependent cell migration (Nakao et al. 2008). Studies show that NCKAP1 drives the actin assembly and polymerization as well as the formation of lamellipodia, which is essential for cell motility and adhesion (Steffen et al. 2004; Teng et al. 2016; Xiong et al. 2019), and this is associated with the development of tumor invasive and metastatic phenotypes. A single-factor analysis of breast cancer showed that the high expression of NCKAP1 was strongly associated with poor metastasis-free survival of breast cancer patients, suggesting that NCKAP1 is an independent prognostic factor of breast cancer (Lomakina et al. 2016). In this study, the invasion and migration abilities of GC cells were significantly up-regulated after overexpression of NCKAP1. By target prediction and dual-luciferase assay, we verified that NCKAP1 was a target gene of miR383-5p, by overexpressing miR383-5p, the expression of NCKAP1 in GC cells was down-regulated, and the increased invasion and migration abilities of GC cells caused by NCKAP1 overexpression were partially reduced. This result is consistent with the above studies, and suggests that NCKAP1 may be involved in distal metastasis of gastric tumors.

However, our study also has its limitations. We only investigated the effect of miR383-5p and its NCKAP1 on the migration and other abilities of GC cells in the in vitro cellular experiments, and its downstream proteins and pathways need to be further investigated. Relevant studies suggested that HSP90 enhanced the stability of NCKAP1 protein in non-small-cell lung cancer (NSCLC) cells, and the overexpression of HSP90 enhanced the accumulation of active MMP9 and the H661 invasion of NSCLC cells. However, this enhancement was attenuated when NCKAP1 was depleted, and HSP90 deletion inhibited the the NCKAP1-mediated NSCLC metastasis in mice (Xiong et al. 2019). The acquisition of invasive and metastatic phenotypes is associated with the loss of genes that promote metastasis as well as the metastasis suppressor genes (Hurst and Welch 2011; Nguyen and Massague 2007). The WASF3 gene has been shown to be a promoter of cell invasion in vitro and metastasis in vivo in different cancer cell types (Teng et al. 2010, 2013). The inactivation of WASF3 in both breast cancer and prostate cancer cells can result in reduced cell migration and invasion (Sossey-Alaoui et al. 2007; Teng et al. 2010). According to recent studies, the NCK/NCKAP1 complex interacted with WASF3, and NCKAP1 was necessary for the WASF3 functions and its invasion regulation (Teng et al. 2016).

Tumor cells can interact with surrounding cells to create an environment that promotes tumor growth and protect the tumor from immune attack (Bissell and Radisky 2001). The extracellular matrix (ECM) affects the tissue and organ structures, as well as the growth of tumor cells (Spencer et al. 2007). The matrix metalloproteinases (MMPs) are ECM proteases that asscociated to carcinogenesis and metastasis(Comoglio and Trusolino 2005). MMPs can be synthesized by tumor cells, but are usually produced by the surrounding stromal cells, including fibroblasts and infiltrating inflammatory cells (Coussens et al. 2002). They can influence the cellular properties such as growth, death and migration, and contribute to invasion, promotion, angiogenesis, and the establishment and growth of metastatic lesions at distant organ sites (Coussens et al. 2002). Sufficient evidences prove that the metastatic ability of tumors is closely related to their production or induced production of basement membrane proteins that degrade the ECM, among which metalloproteinases (MMP) and GC have been better studied. TIMP is a natural inhibitor of MMP, and an imbalance between MMP and TIMP in the extracellular matrix is closely related to tumor infiltration and metastasis. In this study, the in vitro overexpression of NCKAP increased the MMP2 expression and activity in the STAD cells, whereas miR383-5p inhibited the increase in MMP2 expression and activity caused by NCKAP. These results suggest that miR383-5p-NCKAP may affect the motility of stomach adenocarcinoma cells by inducing the change of MMP2 activity and expression.

In conclusion, we proved that miR383-5p can negatively regulate NCKAP1 through direct interaction with the 3’UTR sequence of NCKAP1. Restoration of miR383-5p expression in GC cells can inhibit the invasion and migration abilities of GC cells, whereas overexpression of NCKAP1 can increase the metastatic ability of GC cells. Therefore, overexpression of miR383-5p can suppress the increase in the aforementioned abilities of GC cells induced by NCKAP1 overexpression. It is suggested that the regulation of NCKAP1 by miR383-5p may be involved in the metastasis of gastric tumors. In a word, our data can provide new insights into the search and development of new targets for the treatment of gastric cancer.

## Data Availability

All relevant data are available from the corresponding authors on request.
